# Supplementary material for: Strong-field coherent control of isolated attosecond pulse generation
Source: Nat Commun. 2021 Nov 17;12:6641. doi: 10.1038/s41467-021-26772-0 (PMC8599423; doi:10.1038/s41467-021-26772-0)
Supplement: Supplementary file 1 — Supplementary information [file 41467_2021_26772_MOESM1_ESM.pdf]

SUPPLEMENTARY INFORMATION
Strong-field Coherent Control of Isolated Attosecond Pulse Generation

Yudong Yang[1,2,4], Roland E. Mainz[1,2,4], Giulio Maria Rossi[1,2,4], Fabian Scheiba[1,2], Miguel A. Silva-Toledo[1,2], Phillip D. Keathley[3], Giovanni Cirmi[1,2], and Franz X. Kärtner[1,2*]

[1]*Center for Free-Electron Laser Science, Deutsches Elektronen-Synchrotron DESY, Notkestraße 85, 22607 Hamburg, Germany*

[2]*Physics Department and The Hamburg Centre for Ultrafast Imaging, University of Hamburg, Luruper Chaussee 149, 22761 Hamburg, Germany*

[3]*Research Laboratory of Electronics, Massachusetts Institute of Technology, 77 Massachusetts Ave, Cambridge, 02139 MA, USA*

[4]*These authors contributed equally: Yudong Yang, Roland E. Mainz and Giulio Maria Rossi.*

∗*email: franz.kaertner@desy.de*

Contents

S1 Optical waveform characterization via 2DSI **3**

S2 HHG spectral analysis **6**

S3 HHG photon flux and pulse energy **8**

S4 Attosecond pulse reconstruction **14**

S5 HHG simulations **16**

S6 Data Acquisition Procedure for CEP/RP-scans **25**

S7 References **29**

S1 Optical waveform characterization via 2DSI

Here we report on the synthesized waveform characterization based on the two-dimensional spectral shearing interferometry (2DSI) technique[1]. We performed 2DSI on the two individual pulses (NIR and IR) that compose the waveforms. The 2DSI setup allows one to measure spectral-intensity and spectral-phase of the ultrabroadband pulses of each channel, leaving the corresponding carrier-envelope phases (CEPs), and the relative phase (RP), unknown. Since CEPs of both NIR and IR pulses are linked to that of the common seed, they can be adjusted by controlling the CEP of the seed, in the seeding front end. The pulse-to-pulse CEP variation of the overall synthesized waveform is measured via f-$2f$ interferometry at the synthesis point and stabilized by a feedback loop. The absolute CEP value of the synthesized waveform at the interaction point can be inferred from the observation of CEP-dependent HHG spectra or directly measured via attosecond streaking.

In order to reassemble the synthesized waveforms from the individual pulses we need to determine the CEP as well as the RP (or delay) between the two pulses. The latter can be obtained by broadening the IR pulse spectrum and observe the interference with the NIR pulse. The RP and the "relative delay" among the two pulses are effectively synonyms, since one can convert from one to the other by multiplying by the speed of light in vacuum and dividing by the wavelength at which the RP beating occurs (RP $=$ relative delay $\times\ c/\lambda_{RP}$). We prefer the term RP over "relative delay" since what we observe via the phase meter[2] is indeed the RP extracted from the spectral interference. To stabilize and control the RP, one pulse is delayed with respect to the other via piezo-actuated delay-lines. A more detailed description of the multi-phase meter that simultane-

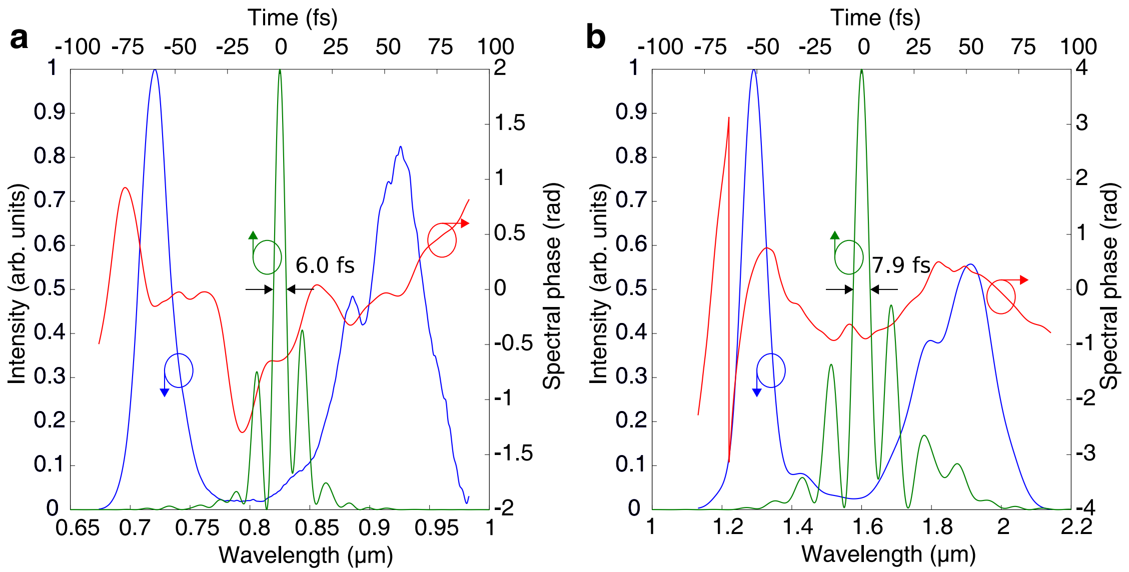

Figure S1: **Pulse temporal characterization with 2DSI.** Spectro-temporal characterization of the NIR **a**, and IR **b**, pulses obtained via 2DSI.

ously detects RP and CEP of the synthesized waveforms can be found in G. M. Rossi *et. al.*[2], while additional information on the phase stabilization and control system can be found in section S6 of this supplementary information. The complete set of waveforms, and the corresponding intensity profiles, that is possible to synthesize can be calculated from the individual pulses in Fig. S1 by numerically varying the CEP and the RP. The RP between both channels is imprinted by adding a linear term on the spectral phase of the IR channel. A zero-order phase is simultaneously added to both NIR and IR channels to mimic the CEP variation. As shown in Fig. S2, these two parameters allow access to a wide variety of intensity profiles and waveforms.

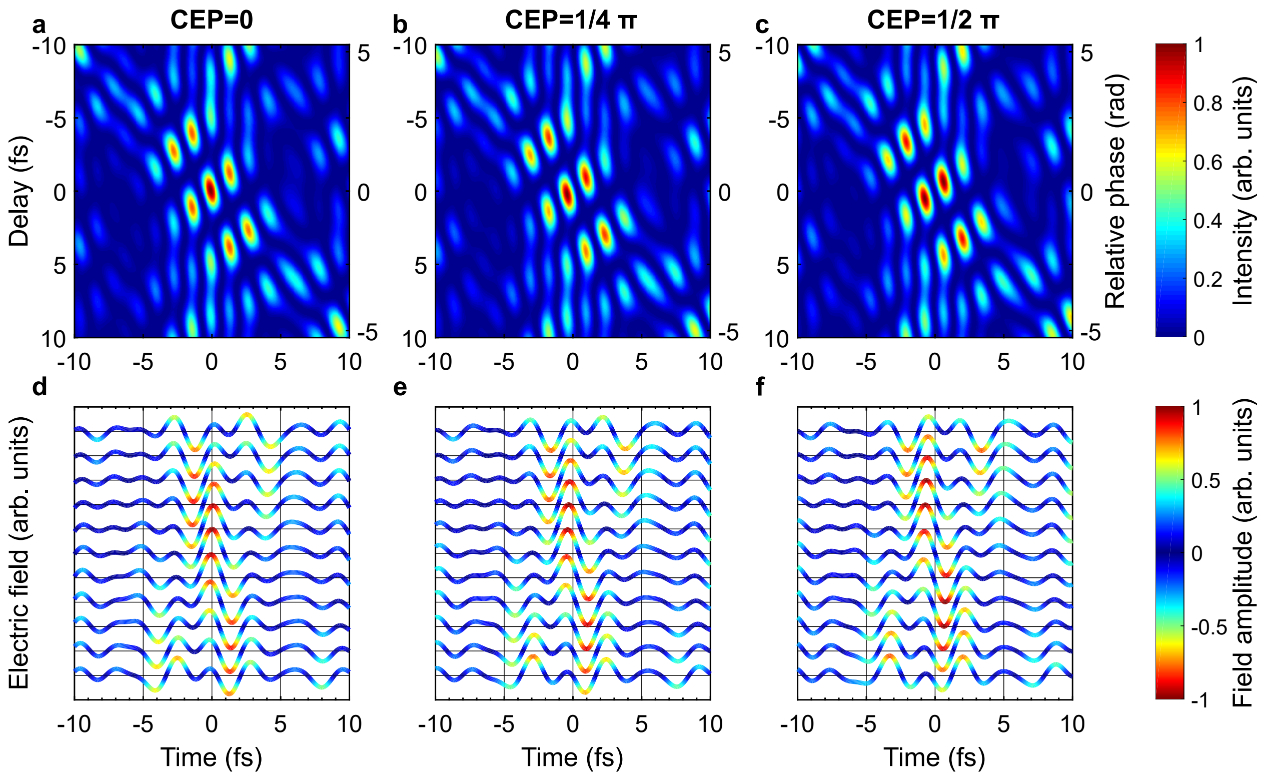

Figure S2: **Numerical relative-phase scan.** Evolution of the intensity profile of the numerically synthesized waveform during a RP scan for **a**, CEP=0, **b**, CEP=$\pi/4$, **c**, CEP=$\pi/2$. **d-f**, Corresponding evolutions of the synthesized field for RP values between -7 rad (bottom) and 7 rad (top) (each step 0.7 rad or ≈ 380 as). Delay $=$ RP $\times\ \lambda_{RP}/c$, $\lambda_{RP} = 990\,\mathrm{nm}$.

S2 HHG spectral analysis

The HHG spectrum was recorded with a McPherson 251MX extreme ultraviolet (XUV) grating spectrometer equipped with an Andor Newton 940 CCD camera. There are two variable-line-spacing (VLS) gratings (300 groove/mm and a 1200 groove/mm) for resolving different spectral ranges ($\approx$ 10 - 60 eV and $\approx$ 60 - 250 eV). The absorption edges of various metallic filters (Al, Be and Zr) were used for photon energy calibration and thereby for the corresponding Jacobian-correction. When measuring broadband HHG emission, the low-energy side of the first-diffraction order (FDO) of the spectrum can overlap with the second (SDO) diffraction order of the high-energy side, resulting in a modified spectral shape. Fig. S3 shows the procedure of removing the SDO contribution when using the 300 groove/mm grating. We start with a narrowband spectrum to easily atttribute spectral features to the FDO and the SDO. For example, as shown in Fig. S3, the two main peaks around 42 eV and 21 eV are the same spectral feature and manifest as the FDO and the SDO of the grating. The SDO-to-FDO signal amplitude ratio is inferred by comparing the SDO and FDO peaks, and assuming the ratio is constant over the entire spectral range. A replica of the spectrum shifted to half the photon energy is then multiplied by the measured SDO-to-FDO amplitude ratio (= 0.2). Finally, by subtracting this photon energy-shifted and attenuated replica from the original spectrum, the SDO contribution to the relevant portion of the spectrum is removed. Any residual contribution is filtered out with a Tukey-window (see Fig. S3b). A similar correction was not required for data acquired with the 1200 groove/mm grating since there were no observable higher order diffraction signals.

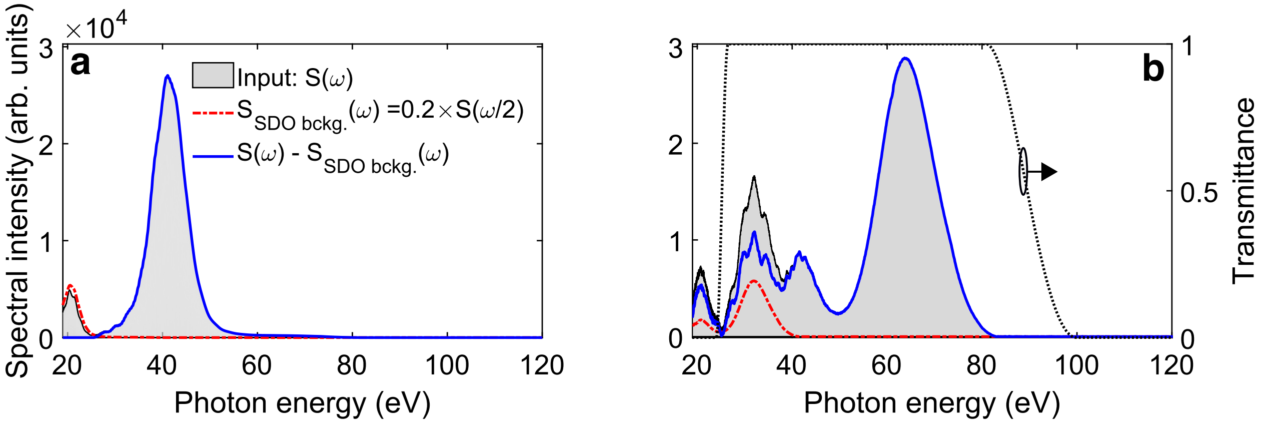

Figure S3: **Second diffraction order (SDO) removal on 300 groove/mm grating.** SDO removal of **a**, narrowband and **b**, broadband spectra. **a**, A narrowband XUV input spectrum (shaded area) showing both its first diffraction (FDO) and SDO, which serves to compute the SDO-to-FDO amplitude ratio. The background due to SDO, (red dashed line) is the product between the SDO factor (0.2) and a replica of input narrowband (**a**) or broadband (**b**) spectra at half the energy. Its subtraction from the input signal yields the SDO-corrected spectra (blue solid line). A Tukey window (black dotted line in **b**) is applied to remove residual contributions to the spectra.

The effect of the CCD quantum efficiency (QE) and grating diffraction efficiency on the shape of the Jacobian-corrected spectra is shown in Fig. S4. For the 300 groove/mm grating, the product of the CCD-QE and the grating efficiency curve is close to constant over the relevant spectral range. Consequently, the shape of the calibrated spectrum is almost identical to the uncalibrated one (see Fig. S4a (i-ii)) and the corresponding spectra presented in the main text (Fig. 2) are only Jacobian-corrected. Meanwhile, for the 1200 groove/mm grating cases (see Fig. S4b (i-ii)), both the CCD and the grating responses are taken into account in Fig. 2 b, e, f and i of the main text.

S3 HHG photon flux and pulse energy

Measurement of the photon flux and pulse energy based solely on the XUV CCD detector can often carry several errors. The precise spectral response of each reflective or transmissive elements (e.g., metallic filters, gratings, etc.) needs to be known. For instance, due to oxidation, manufacturing imperfection and/or contamination these can differ to what is reported in published databases [3–5]. Careful alignment and beam pointing stability through apertures coupling the beam into the spectrometer also has to be assured, owing to the fact that small deviations in the HHG beam pointing can lead to truncation of the beam and thereby to a considerably different energy throughput. Furthermore, the latter is of utmost importance, since the divergence of the XUV/soft X-ray beam depends on the precise HHG generation conditions (i.e., driving synthesized waveform, gas pressure, gas-cell position, etc.) and can result in a clipped beam reaching the CCD. Thus, the lack of inspection on these details can easily lead to a significant underestimation of photon number and pulse energy values.

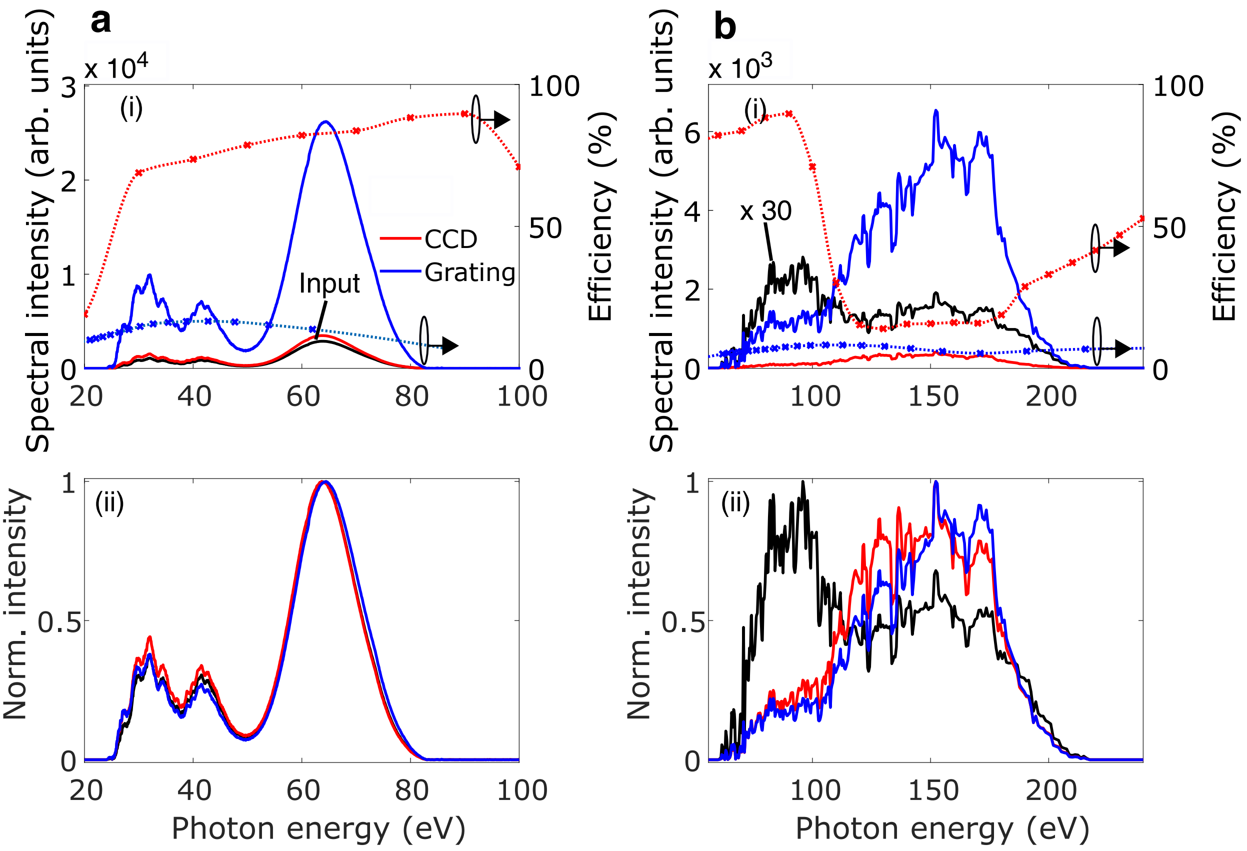

Figure S4: **Influence of CCD and grating response on HHG spectra.** HHG spectra of **a** argon (350 mbar) and **b**, neon (300 mbar) using the 300 groove/mm and 1200 groove/mm gratings, respectively. **a** – **b** (i), the black, red and blue curves represent the raw, CCD-corrected and CCD &grating-corrected spectrum, respectively (left axis). The CCD response (red crosses), grating diffraction efficiency (blue dashed crosses), and the corresponding splines (dashed lines) are shown as well (right axis). In **a** (i), the grating diffraction efficiency curve has been extrapolated up to 86 eV. In **b** (i), the black curve has been multiplied by 30 for visibility. **a** – **b** (ii), Spectra from (i) normalized separately.

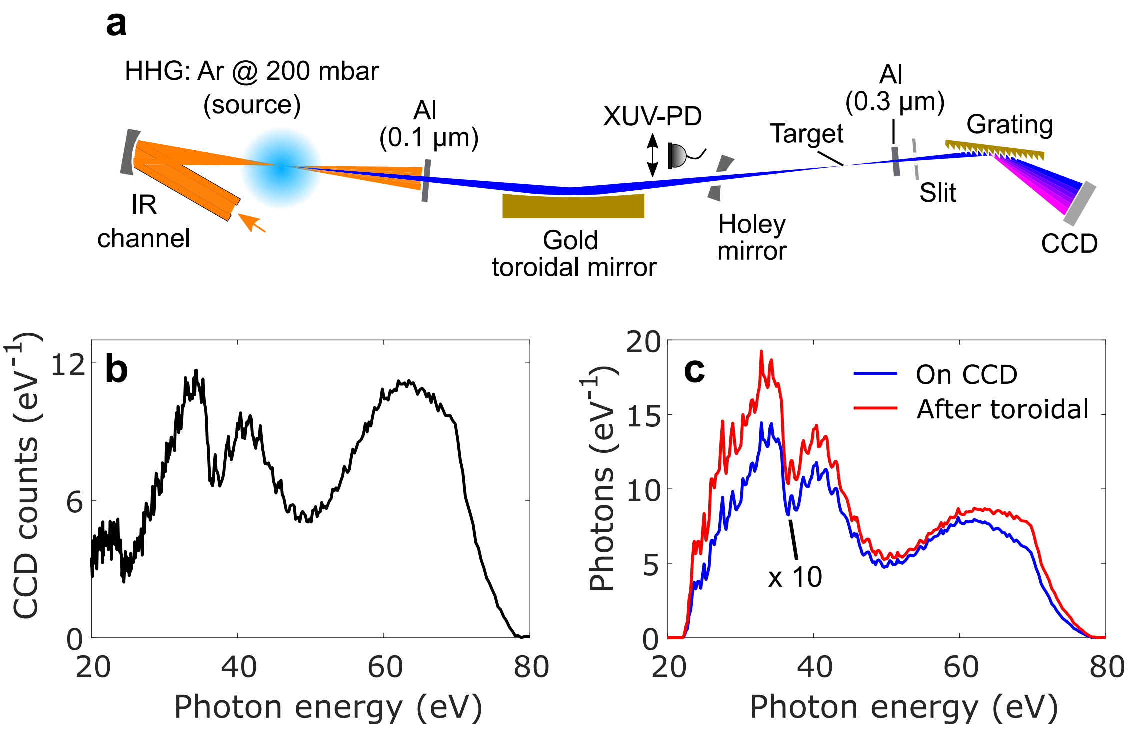

Figure S5: **HHG photon number reference measurement.** **a**, Experimental scheme. HHG was driven by the IR channel on an Ar-target at 200 mbar (thickness ≈ 2 mm). A calibrated photodiode (Opto Diode, SXUV100) was placed after a 0.1 μm-thick Al foil and a gold-coated toroidal mirror. Harmonics were spectrally resolved with a variable line spacing grating (300 lines/mm) and a CCD camera (Andor Newton 940) after passing through a holey mirror, a 0.3 μm-thick Al filter and a slit. **b**, Jacobian- and background-corrected CCD signal, S_{CCD}. The laser repetition rate (1 kHz) and CCD integration time (1 s) was considered. **c**, Number of incident photons on the CCD, n_{CCD}, (blue curve). The CCD sensitivity ($\sigma = 1$), quantum efficiency (see Fig. S4) and generated electron-hole pairs were included (c.f. Eq. 1). The second diffraction order was removed as described in Fig. S3 and the final curve was multiplied by a factor of 10 for visibility. The number of photons after the toroidal mirror (red curve), n'_{CCD}, results after removing the grating (see Fig. S4) and the 0.3 μm-Al filter spectral responses (c.f. Eq. 2).

A calibrated XUV photodiode (XUV-PD) can give a more accurate measurement of these values, provided that the spectral shape of the pulse is known. As in our experiments we acquired hundreds of spectra, alternating between the CCD and the XUV-PD for each spectrum is impossible. Therefore, we measured the number of photons of a reference HHG spectrum employing both a photodiode as well as a XUV spectrometer. The ratio between the two measurements was used as a calibration factor to estimate the photon flux associated with the attosecond pulses based in their spectra, shown in Fig. 4 of the main article. The procedure used to derive the calibration factor is described in the following paragraphs.

Fig. S5a shows the experimental scheme we used to measure the reference HHG photon number. The IR channel was focused ($f = 375$ mm) onto the gas-cell filled with 200 mbar of Ar. An aluminum filter with a thickness of $0.1\ \mu$m transmitted the emitted XUV harmonics and blocked the co-propagating IR field. The harmonics were then guided by a gold-coated toroidal mirror towards the CCD-based spectrometer located after a holey mirror, an aluminum foil (0.3 μm thickness) and an entrance slit. The XUV-PD was mounted on a motorized translation stage that can be inserted into the beam path right after the toroidal mirror. The XUV-induced photocurrent was measured with an oscilloscope after amplifying and converting it into a voltage with an operational amplifier (AXUV100HYB1V from IRD Inc.) having a feedback resistor of 10 GΩ. With the XUV spectrometer, we acquired the HHG signal shown in Fig. S5b. With the responsivity curve of the photodiode provided by the manufacturer and using a central energy of 50 eV (centre of mass of the spectral intensity) we estimated a total of $N_{\mathrm{PD}} = 5.63 \times 10^4$ photons per pulse.

For estimating the number of photons after the gold toroidal mirror via the CCD measurement, we first compute the number of incident photons on the CCD from the CCD signal S_{CCD} (see Fig. S5b) as

$$n_{\text{CCD}}(\omega) = \frac{S_{\text{CCD}}(\omega) \cdot \sigma}{\eta_{\text{QE}}(\omega) \cdot n_{\text{e-h}}(\omega)} \tag{1}$$

where σ is the sensitivity in electrons per CCD count, η_{QE} is the CCD quantum efficiency (see Fig. S4) and $n_{\text{e-h}}(\omega) = \hbar\omega/3.65$ is the number of electron-hole pairs freed per incident photon with energy $\hbar\omega$[5,6]. Next, we calculate the number of photons at the location of the XUV-PD, i.e., after the gold toroidal mirror (without considering any transmission drop caused by the holey mirror and input slit) by further taking into account the grating efficiency η_{g} and filter transmission t_{f}, i.e.,

$$n'_{\text{CCD}}(\omega) = \frac{n_{\text{CCD}}(\omega)}{\eta_{\text{g}}(\omega) \cdot t_{\text{f}}(\omega)} \tag{2}$$

It is necessary to consider the transmission of the grating spectrometer and of the filter in front of the spectrometer since different gratings and different filters were used. Fig. S5c displays the n_{CCD} (blue curve) and n'_{CCD} (red curve) as function of the photon energy. Accordingly, the total number of photons per pulse is determined as

$$N_{\text{CCD}} = \int n'_{\text{CCD}}(\omega) d\omega, \tag{3}$$

which outputs a value of $N_{\text{CCD}} = 475$ photons per pulse. As both the photodiode and CCD measurement need to yield the same value, we force $N_{\text{CCD}} = N_{\text{PD}}$, which results in a calibration factor given by $\alpha_0 := N_{\text{PD}}/N_{\text{CCD}} \approx 118$. Finally, the spectrally-resolved number of photons on target is $n_{\text{target}} = \alpha_0 \cdot n'_{\text{CCD}}$ and, thus, the total number of photons per pulse is evaluated as

$$N_{\text{target}} = \int n_{\text{target}}(\omega) d\omega, \tag{4}$$

Equivalently, the pulse energy on target is

$$U_{\text{target}} = \int n_{\text{target}}(\omega) \cdot (\hbar\omega) d\omega, \tag{5}$$

Table 1 contains the photons per shot and pulse energies of the spectra shown in Fig. S5 and in Fig. 4 of the main text. Values were computed using Eqs. 4-5. For retrieving these values at the HHG source point, Eq. 2 needs to be additionally divided by the toroidal mirror reflectivity (at 4° AOI) and, if applicable, any filter transmission. Previously reported number of photons and pulse energy values are also exhibited for comparison.

	On target		At source	
**Case**	**Photons/pulse**	**Pulse energy (pJ)**	**Photons/pulse**	**Pulse energy (pJ)**
IR channel (Fig. S5)	5.63×10^4	0.41	8.71×10^4	0.62
Fig. 4a	7.15×10^7	466.78	8.96×10^7	585.04
Fig. 4b	9.24×10^6	79.35	1.39×10^7	117.64
Fig. 4c	1.04×10^6	10.58	1.30×10^6	13.21
Fig. 4d	1.15×10^6	13.15	1.41×10^6	16.09
Ref. [7]	5.30×10^8	2.10×10^3	-	-
Ref. [8]	3.33×10^4	-	-	500.00
Ref. [9]	-	-	5.00×10^5	-
Ref. [10]	1.00×10^7	300.00	-	-

Table 1: **Estimated XUV photon numbers and pulse energies.** Total number of photons and pulse energies of spectra presented in Fig. 4 of the main text. Values were computed on target and at the source, using the scaling factor α_0 and Eqs. 4 – 5 .

S4 Attosecond pulse reconstruction

For the attosecond pulse reconstruction the time-axis of the time-of-flight (TOF) data was initially converted into the corresponding kinetic-energy axis. This calibration was realized by matching the HHG comb peaks (generated in argon) measured with the XUV spectrometer to the peaks measured by the TOF spectrometer (with neon as streaking gas), and by fitting them with a parabola.

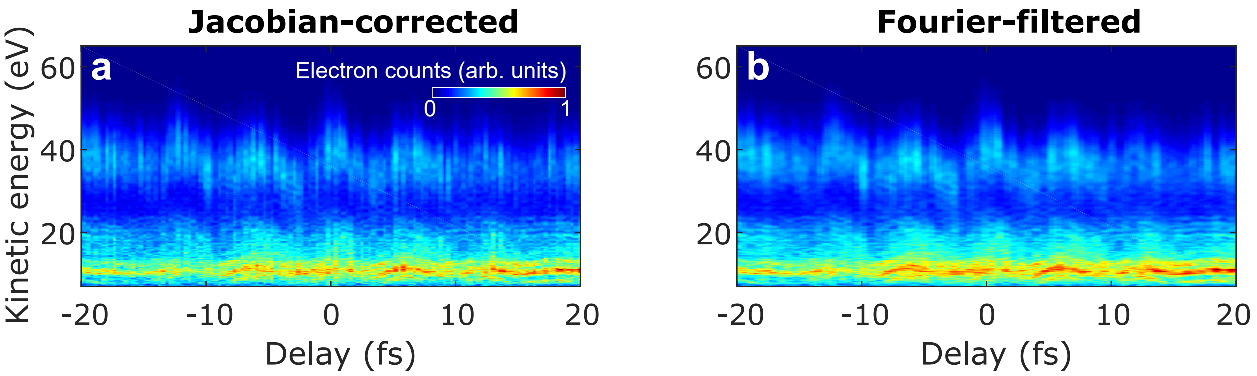

Figure S6: **Fourier-filtering of photoelectron spectrograms.** **a**, Photoelectron spectrogram after kinetic energy calibration and Jacobian-correction, and **b**, after additional filtering of high-frequency noise (> 950 THz).

The Jacobian correction factor was then applied to calibrate the spectral intensity. The Shirley background, resulting from low-kinetic energy electrons (below $\approx$ 10 eV), was also subtracted from photoelectron spectra. Lastly, with the purpose of minimizing inaccuracies in the streaking trace reconstruction, the intrinsic high-frequency measurement noise along the delay axis was eliminated by filtering out frequencies above the second-harmonic of the highest frequency in the synthesized spectrum, that is ≈ 950 THz (see Fig. S1a and Fig. S6).

After applying the procedure described above, the data-sets are ready to be reconstructed. To this end, we used the Volkov-Transform Generalized Projections Algorithm (VTGPA)[11]. The algorithm includes the complex-valued and energy-dependent dipole matrix elements of neon (p-electron orbital; $I_\mathrm{p} \approx 21.7$ eV). The initial guess of the XUV-pulse was represented by a Gaussian envelope, with its central energy being inferred from the input spectrogram. The vector potential of the streaking field was represented as the product of an envelope function and a carrier wave, $A(t) =$

$A_N(t)\cos\alpha(t)$. The envelope function $A_N(t)$ was described as a cubic-spline interpolation of N points while the carrier wave as a polynomial expansion with k coefficients, namely, $\alpha(t) = \alpha_0 + \alpha_1 t + \alpha_2 t^2 + ... + \alpha_k t^k$. We found that to reconstruct the envelope function of the synthesized streaking fields up to $N = 60$ and $k = 3$ points were necessary (see Fig. S7). The time sampling step size in the reconstruction was set to be 4-5 times shorter than the main XUV oscillation period.

Fig. S8 compares the VTGPA-retrieved streaking fields from Fig. 4 of the main text, with the fields retrieved by simply following the evolution of the center of mass (COM)[12] of the photoelectron spectra along the delay axis. The close resemblance between the two extracted fields and the small residual error between the original and reconstructed traces suggest a good fidelity of the measurements and reconstructions. Furthermore, for all attosecond streaking traces, the overall residual mean square error (MSE) between measured and reconstructed spectrograms, after 300 iterations of the algorithm, was found to be in the 10^{-3} range or lower.

S5 HHG simulations

Single-atom response simulations were performed based on the Strong Field Approximation (SFA) within the Lewenstein theory[13] using the open-source code, HHGmax[14,15]. Static ionization rates[16] are used to compute the ground state depletion and hydrogen-like dipole matrix elements are assumed for the dipole moment $d(t)$ calculation. Long trajectory emissions are usually suppressed during propagation due to phase mismatch caused by their strongly intensity-dependent phase[17,18]

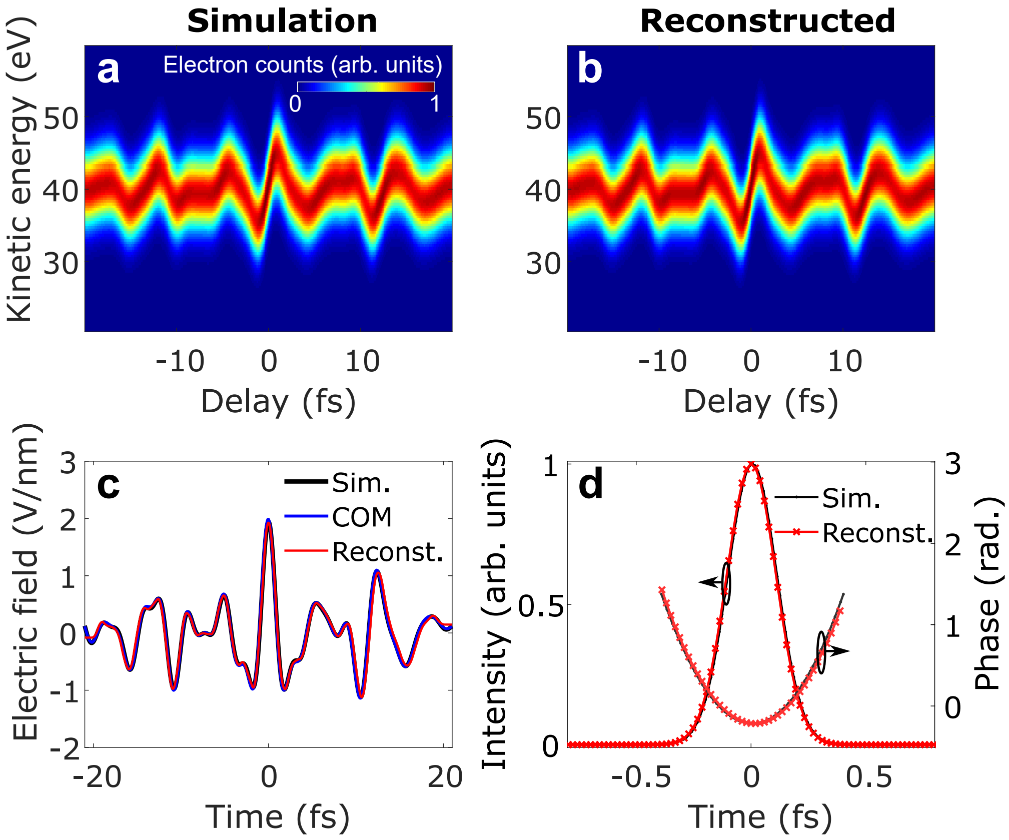

Figure S7: **Attosecond streaking simulation and reconstruction.** **a**, Simulated photoelectron spectrogram. The streaking field was built upon the superposition of the actual NIR and IR channel temporal profiles. The isolated attosecond XUV pulse was modelled as a Gaussian envelope centred at 60 eV with a quadratic frequency chirp. **b**, Reconstructed streaking trace after 300 iterations. The number of cubic-spline interpolation points was set to $N = 60$. **c**, Comparison between the simulated (black), centre-of-mass (COM)-retrieved (blue) and reconstructed (red) electric fields. **d**, Simulated (solid black line) and reconstructed (red crosses) intensity profile (left axis) and phase (right axis) of isolated attosecond pulse.

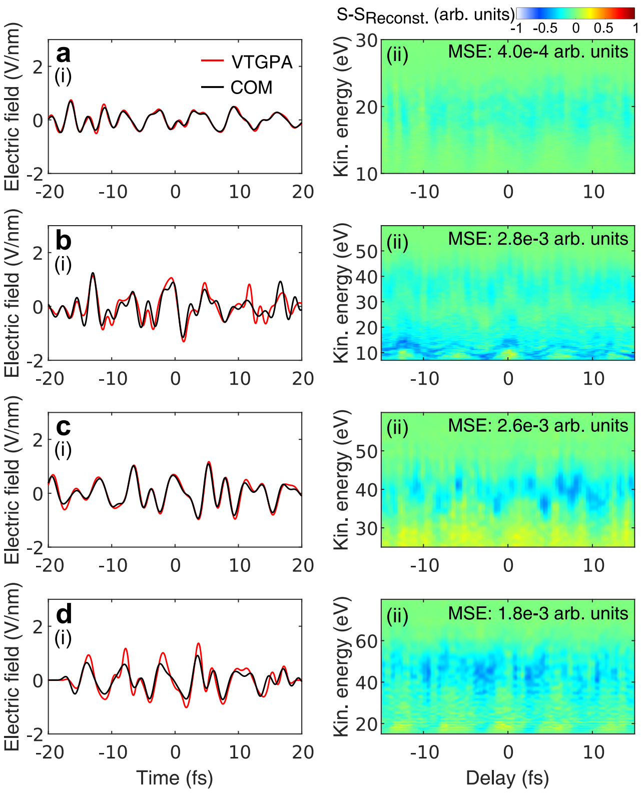

Figure S8: **Retrieved streaking waveforms and reconstruction error. a-d**, (i) centre of mass (COM)- and VTGPA-retrieved streaking electric fields from photoelectron spectrograms shown in Fig. 3 of the main text (with the same label ordering). In (i) all frequencies above $\approx$ 475 THz (highest observed frequency in the synthesized spectrum) were set to zero in transients retrieved with the COM method. (ii) Difference between the intensity-normalized experimental and reconstructed spectrograms. The residual mean squared error (MSE) after 300 iterations is shown on the upper right corner in each case.

and due to their spatial divergence[19], thus they were excluded during the Lewenstein integration and only the contribution from short trajectories was considered. The electric field $E_L(t)$ of the input driving waveforms was obtained from the temporal superposition of the measured NIR and IR channel electric fields, with the RP and the CEP being numerically varied as described in Sec.S1. The peak intensity was set as 2.5×10^{14} W/cm^2 when the RP and the CEP are both set to 0 rad. The IR channel intensity was chosen to be about four times the NIR channel intensity. Here, we did not use the retrieved IR waveforms from attosecond streaking measurements as simulation inputs. The reason is that the waveform replica used in the streaking experiment is not identical to the one driving the HHG, due to the non-uniform attenuation caused by the variable aperture and the holey mirror. Therefore, we chose to observe how the IAP parameters changes with different synthesis parameters (RP, CEP) and compare with theory calculations computed with a large set of possible waveforms (based on 2DSI measurements superimposed with different synthesis parameters).

To gain insight regarding electron trajectories under the influence of the distinct tailored waveforms, Newton's equation of motion, $F_L(t) = -eE_L(t)$, for an electron $-e$ accelerated by the laser field $E_L(t)$, were numerically integrated assuming that the electron is born at the ionic core with zero initial velocity. Electron trajectories not returning to their origin (not recombining) and those with a negative chirp (long trajectories) were disregarded and only short recombining trajectories were retained. To estimate their relevance on HHG emission, the attenuation level of trajectories in Fig. 5 c-e of the main text was calculated based on the ionization rate value at their birth time. The ionization rate was derived from the static ionization model as in the semi-classical calculations.

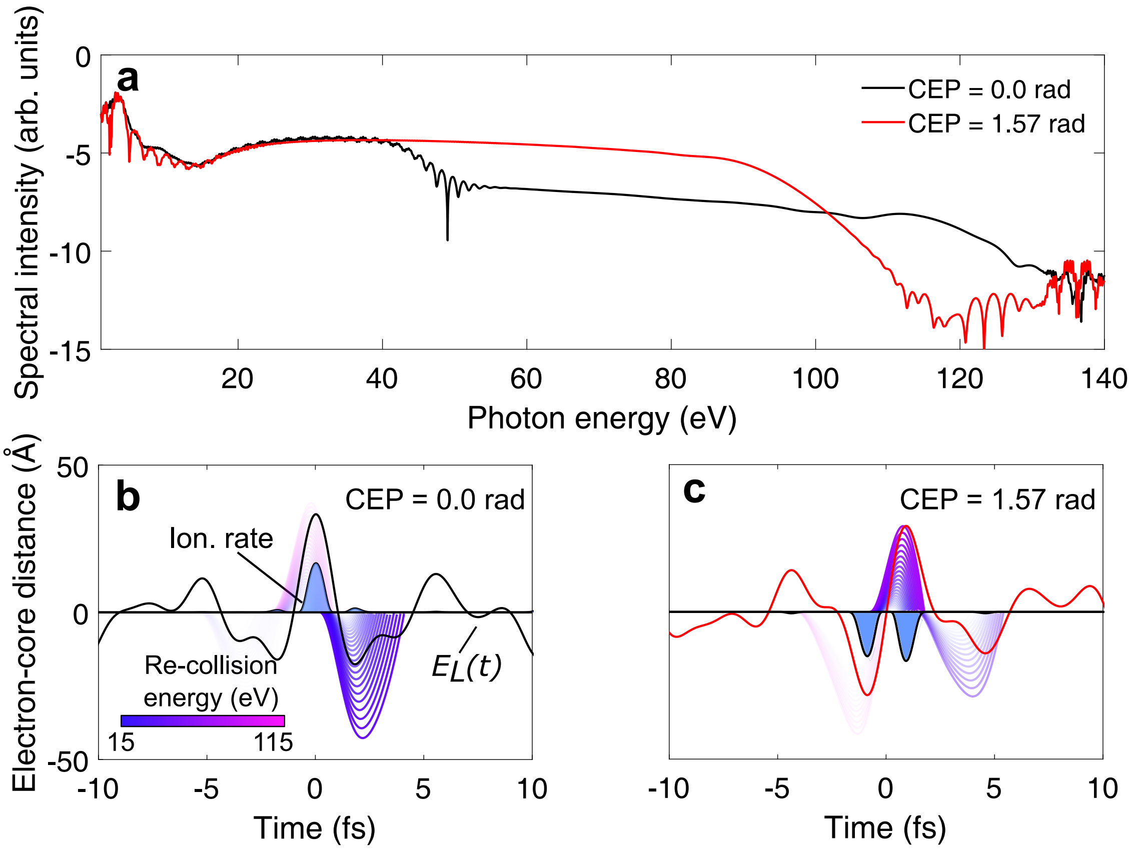

Figure S9: **HHG simulations for argon.** **a**, Single-atom response simulations from Ar driven by tailored optical waveforms with CEP = 0.0 rad and CEP =1.57 rad (π/2). The synthesized driving transient $E_L(t)$ results from the coherent superposition of the NIR and IR channels assuming no relative delay/phase (RP = 0.0 rad) between them. Simulations are based on the Lewenstein model and consider short trajectories only. The peak intensity of the driving waveform is 2.0×10^{15} W/cm^2 with CEP = 0.0 rad and RP = 0.0 rad. Classical electron short-trajectories (purple) for different optical waveforms (black solid lines) with **b**, CEP = 0.0 rad and **c**, CEP = 1.57 rad ($\hat{=}\ \pi/2$). The trajectory color-coding is based on the re-collision energy and the transparency level on the ionization rate (blue shaded area) at the trajectory birth time.

Fig. S9a shows HHG spectra resulting from a single argon atom (I_p = 15.75 eV) for two specific CEP values (0 rad and 1.57 rad). The spectrum was calculated as the absolute square of the high-harmonic field, i.e. $|E_s(\omega)|^2$, with $E_s(\omega) = \omega^2 \cdot d(\omega)$, where ω is the angular frequency and $d(\omega)$ the frequency-dependent dipole moment. For CEP = 0.0 rad, cut-offs around 40 eV and 100 eV result from recombining trajectories released by the most intense (main) and the preceding half-cycles, respectively (see Fig. S9b). The main half-cycle leads to the highest ionization rate and thereby to the first plateau (spanning 20 - 40 eV) with higher yield. Spectral fringes arise due to interference between photons generated in the main and the following half-cycle. In contrast, for CEP = 1.57 rad, a single cut-off around 100 eV and a broad, smooth plateau is observed. In this case, electrons are more efficiently released by the preceding half-cycle and get an increased acceleration by the main half-cycle, yielding the higher-yield plateau. The probability of the second recombination is significantly reduced due the extended electron excursion time (more significant quantum diffusion) in the following half-cycle.

A detailed simulation of propagation effects (including phase-matching)[18,21] occurring during HHG driven by our sub-cycle waveforms goes beyond the scope of the present work. Nevertheless, the simulated single-atom HHG emission is propagated through the gas target by including the linear absorption and refraction of a 1D column of gas as $E_s(\omega)\exp(-ik(\omega)L)$, where $k(\omega) = \omega/c \cdot (n(\omega) - i\beta(\omega))$ is the wave vector, $n(\omega)$ is the real index of refraction, $\beta(\omega)$ the extinction coefficient and c the speed of light. Fig. S10a shows the transmittance and refractive index of argon at 150 and 300 mbar and assuming a medium length of L = 2 mm. Strong absorption and dispersion in argon[20] occurs for photon energies below $\approx$ 40 eV. The propagated energy-

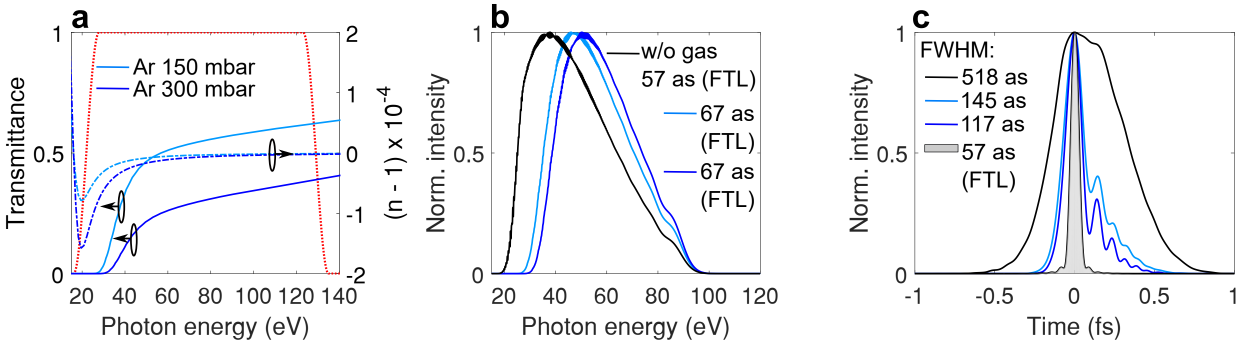

Figure S10: **Attosecond pulse reshaping after propagation through argon.** **a**, Transmittance (solid lines, left axis) and refractive index (dashed lines; right axis) of argon at 150 mbar and 300 mbar[20]. A Tukey window (red dashed line) removing energies below 16 eV and above 135 eV is used to filter out low-order harmonics and to remove numeric noise at high energies. Normalized spectral **b**, and temporal **c**, intensity of attosecond bursts before (black solid lines) and after propagation through 150 and 300 mbar of argon gas (blue solid lines). Fourier transform limited (FTL) and actual pulse durations (FWHM) are displayed for each case in **b** and **c**. The HHG was generated via a driver waveform with RP = 0.0 rad and CEP = 1.57 rad (Fig. S9c). The shaded area in **c** corresponds to the FTL temporal profile without the gas response.

and time-dependent intensity profiles of the emitted XUV radiation are shown in Fig. S10b and c, respectively. A Tukey window was applied to the propagated field to remove energies below 16 eV and above 140 eV. The normalized intensity profiles are shown in Fig. S10b to exhibit the spectral envelope reshaping due to absorption. As a result of the strong absorption for low photon energies and decreasing absorption for high photon energies, pressures between 150 and 300 mbar lead to a blue-shift of the centre wavelength while keeping similar spectral bandwidths (67 as FTL). Absorption below 40 eV and negative dispersion within this photon energy range reduces the FWHM pulse duration of the emitted attosecond burst from 518 as of neglecting the gas response down to 117 as with 300 mbar of argon (see Fig. S10c).

In the attosecond streaking measurements shown in the main text, a 100-nm thick aluminum foil was employed to block the HHG driving field. We explored the effects of the aluminum foil on the attosecond bursts by introducing its absorption and dispersion[22,23] (see Fig. S11a) in the same way as in the gas target case. Fig. S11 b shows the influence of a 100-nm thick aluminum foil on a XUV spectrum reaching energies slightly above 80 eV and with a FTL pulse duration of 90 as (see Fig. S11c). This narrower spectrum (compare with Fig. S10) was obtained by setting the RP and the CEP to 5.7 rad (3 fs) and 1.57 rad, respectively. The absorption and dispersion due to 300 mbar, 2 mm-thick column of argon were also included in the simulation. Within this spectral range, the response of the aluminum foil only introduces a negligible difference ($\approx$ 2 as) to the pulse duration.

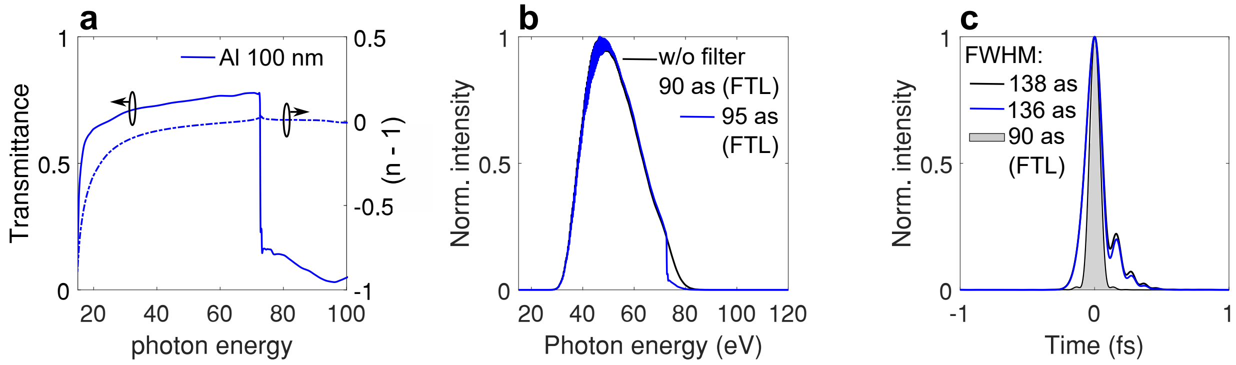

Figure S11: **Attosecond pulse reshaping after propagation through an aluminum foil a**, Transmittance (solid line, left axis) and refractive index (dashed line; right axis) of a 100-nm thick Al foil[22,23]. Normalized spectral **b**, and temporal **c**, intensity of the attosecond burst without (black solid line) and with the aluminum filter response (blue solid line). The Fourier transform limited (FTL) temporal profile corresponding to the unaltered spectrum is displayed in **c** (shaded area). The absorption and refraction of a 2 mm-thick argon gas target at 300 mbar was also included when the aluminum foil was applied.

S6 Data Acquisition Procedure for CEP/RP-scans

The synthesis parameters (CEP, RP) are measured and actively stabilized by an FPGA-based system which generates feedback signals controlling multiple delay lines in the parametric waveform synthesizer. On top, a graphical user interface on the control PC is providing more complex measurement sequences via scripts commanding the FPGA-system. The main detector consists of a home-made dual-phase meter measuring two spectral beat signals (f-f and f-$2f$) derived from the beam at the secondary beam-combiner output (main beam directed to experiment) [24]. The two beat signals overlap spectrally but are set to be at different beat-frequencies allowing separation of the two signals. To this end, the measured single-shot spectra are Fourier transformed and the two corresponding phases are extracted and unwrapped. One of these two phases represents the RP and the other one the sum of CEP and RP. These phase values are fed to a PI-feedback system giving distributed feedback to piezo-driven actuators in our CEP-stable front-end (affecting the CEP) and in one of the synthesizer channels (affecting the RP). Those fast actuators allow to stabilize and control the phases in a limited range within less than 2 laser shots of latency (2 ms). The CEP can be modulated by up to 2 cycles (4π), which is sufficient to perform a full CEP scan due to its 2π-periodicity. For the RP this 2-cycle range is too limited, for that reason a long-range delay line additionally allows to displace the RP by hundreds of cycles (stage-travel: 25 mm). A long-range RP-scan (see Fig. S12) is performed by modulating the locked CE-phase over a 1.75 cycle range with an asymmetric saw-tooth function.

After two of those CEP-scan cycles, the RP-set-point is incremented step-wise by $\pi/4$. This scan-

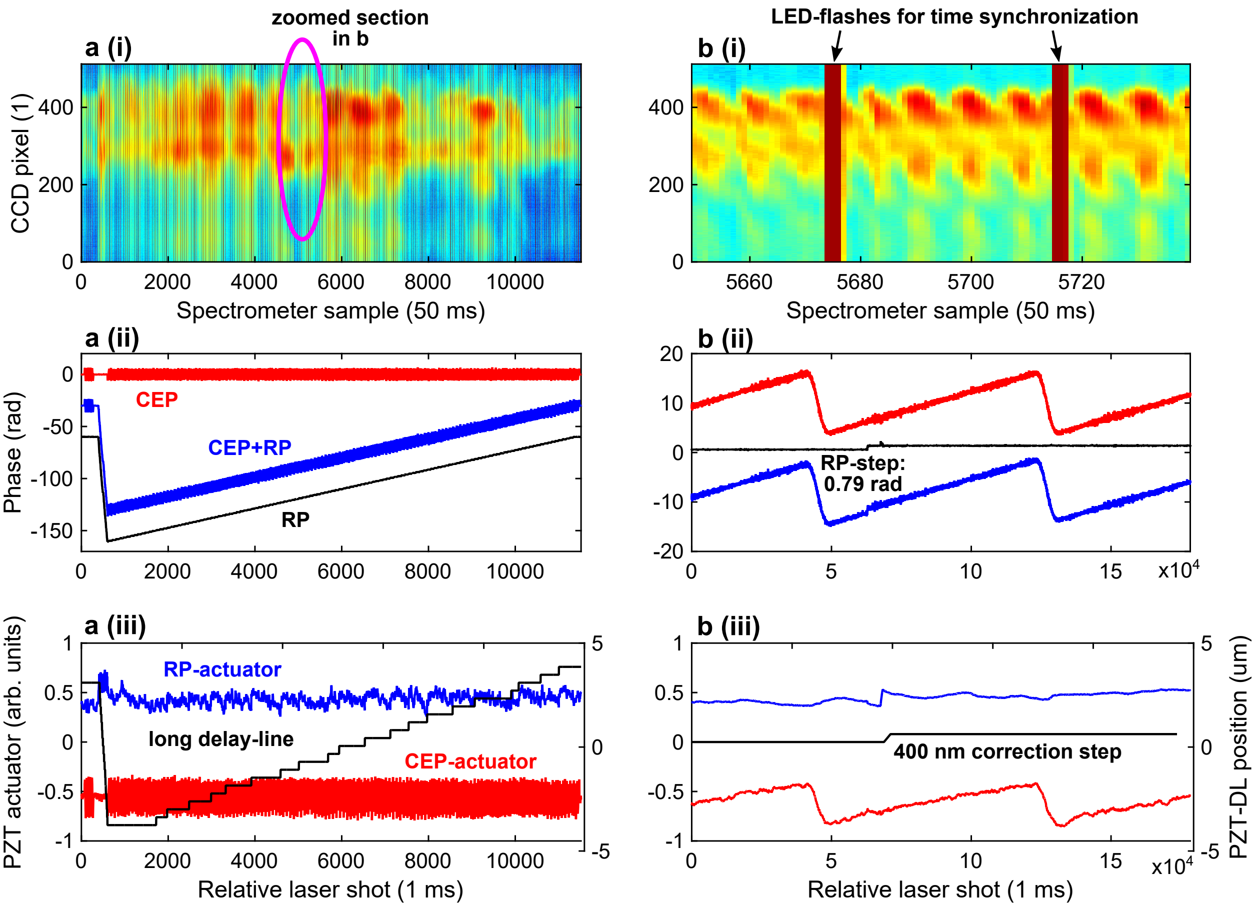

Figure S12: **Synthesizer Control Data for a CEP-RP-scan sequence: a** (i-iii), shows the time-synchronized records of a complete CEP-RP scan, **b** (i-iii), shows a zoomed-in section of **a**. The HHG-spectrogram (i) is recorded while the PWS control system performs phase-scans. The CEP is constantly modulated (ii, red) while the RP is incremented step-wise after two CEP-modulation cycles (ii, black). The measured phases (CEP in red, CEP+RP in blue) are actively stabilized by fast PZT-actuators (iii, red/blue). The RP-setpoint is scanned over a range wider than the RP-actuator allows. This actuator is brought back to its centre position by correcting with long delay-line (**b** iii, black).

ning procedure of the 2D synthesis parameter space is similar to the raster scan of a cathode ray tube display. During such a scan, the control computer observes the position of the fast RP-piezo actuator and, if displaced further than 1/4 of the dynamic range, brings it back to the centre position by moving the long-range delay line. Meanwhile the HH-emission from these synthesized waveforms is continuously recorded with our XUV spectrometer. The linear ramp of the CEP modulation extends over 90% of its period and during the short return time a LED driven by our feedback system flashes into the spectrometer, imprinting a time grid and absolute time markers directly onto the HHG-spectral trace.

In post-processing, this allows us to synchronize the synthesis parameters corresponding to every laser shot with a certain recorded HHG-spectrum. Therefore, the merged data-set contains our synthesis parameters (CEP and RP) and the corresponding response of the HHG. The measured CE-phase here can be wrapped due to its 2π-periodicity. A resorting algorithm then sorts every recorded spectrum onto a bin of a CEP-RP matrix. While the bin-size for the RP is set by the discrete step-size in the sequence controller, the CEP is continuously scanned and put on a grid of π/8 wide bins, optimizing both the number of spectra in each bin, as well as only grouping HHG-responses for very similar synthesized waveforms. The resorted data matrix is checked for consistency by comparing the spectra sorted in each bin for similarity of their shape. While a certain overall XUV-intensity fluctuation is observed (and expected), the spectral shape shows a very good reproducibility. Each bin contains 4-8 spectra and the mean spectrum is calculated. These resorted spectra undergo calibration of the spectral characteristics of our spectrometer (see Section S2) and yields the basis for the data presented. A GUI allows to interactively browse the

4-dimensional data-set and to create constant-CEP and constant-RP cuts through the data (see Fig. S13).

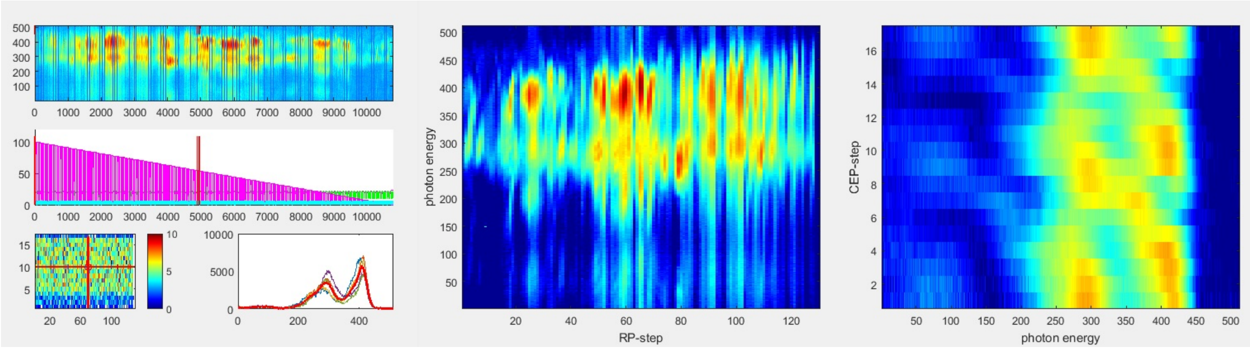

Figure S13: **GUI for data resorting and extraction. a** (i-iv), raw HHG-spectra (i), corresponding phase and gating functions (ii), Spectra-density in each phase bin (iii) and all spectra sorted in one phase-bin (iv). **b,c** shows a cut through constant CEP and constant RP (corresponding to red markers in **a**).

S7 References

1. Birge, J. R., Ell, R. & Kärtner, F. X. Two-dimensional spectral shearing interferometry for few-cycle pulse characterization. Optics Letters **31**, 2063 (2006).

2. Rossi, G. M. et al. Sub-cycle millijoule-level parametric waveform synthesizer for attosecond science. Nature Photonics **14**, 629–635 (2020).

3. The Center for X-ray Optics. X-ray interactions with matter, http://henke.lbl.gov/.

4. Henke, B., Gullikson, E. & Davis, J. X-ray interactions: photoabsorption, scattering, transmission, and reflection at E=50-30000 ev, Z=1-92, Atomic Data and Nuclear Data Tables **54**, 181–342 (1993).

5. Rading, L. An Intense Attosecond Light Source - from Generation to Application. Ph.D. thesis, Lund University (2016). Pages:41-60.

6. Hädrich, S. et al. High photon flux table-top coherent extreme-ultraviolet source. Nature Photonics **8**, 779–783 (2014).

7. Ferrari, F. et al. High-energy isolated attosecond pulses generated by above-saturation few-cycle fields. Nature Photonics **4**, 875–879 (2010).

8. Goulielmakis, E. et al. Single-Cycle Nonlinear Optics. Science **320**, 1614 (2008).

9. Li, J. et al. 53-attosecond X-ray pulses reach the carbon K-edge. Nature Communications **8**, 186 (2017).

10. Ossiander, M. et al. Attosecond correlation dynamics. Nature Physics **13** (2016).

11. Keathley, P. D., Bhardwaj, S., Moses, J., Laurent, G. & Kärtner, F. X. Volkov transform generalized projection algorithm for attosecond pulse characterization. New Journal of Physics **18**, 073009 (2016).

12. Gagnon, J. & Yakovlev, V. S. The direct evaluation of attosecond chirp from a streaking measurement. Applied Physics B **103**, 303–309 (2011).

13. Amini, K. et al. Symphony on strong field approximation. Reports on Progress in Physics **82**, 116001 (2019).

14. Hoegner, M. https://github.com/Leberwurscht/HHGmax (accessed on 3 Feb. 2021).

15. Högner, M., Tosa, V. & Pupeza, I. Generation of isolated attosecond pulses with enhancement cavities—a theoretical study. New Journal of Physics **19**, 033040 (2017).

16. Tong, X. M. & Lin, C. D. Empirical formula for static field ionization rates of atoms and molecules by lasers in the barrier-suppression regime. Journal of Physics B: Atomic, Molecular and Optical Physics **38**, 2593–2600 (2005).

17. Salières, P., L'Huillier, A. & Lewenstein, M. Coherence control of high-order harmonics. Phys. Rev. Lett. **74**, 3776–3779 (1995).

18. Gaarde, M. B., Tate, J. L. & Schafer, K. J. Macroscopic aspects of attosecond pulse generation. Journal of Physics B: Atomic, Molecular and Optical Physics **41**, 132001 (2008).

19. Bellini, M. et al. Temporal coherence of ultrashort high-order harmonic pulses. Phys. Rev. Lett. **81**, 297–300 (1998).

20. Chantler, C. et al. X-Ray Form Factor, Attenuation and Scattering Tables (version 2.1). National Institute of Standards and Technology, Gaithersburg, MD. (2005). [Online] Available: http://physics.nist.gov/ffast [2021, May 27].

21. Schötz, J. et al. Phase-matching for generation of isolated attosecond xuv and soft-x-ray pulses with few-cycle drivers. Phys. Rev. X **10**, 041011 (2020).

22. Polyanskiy, M. N. Refractive index database. [Online] Available: https://refractiveindex.info [2021, May 27].

23. Rakic, A. D. Algorithm for the determination of intrinsic optical constants of metal films: application to aluminum. Appl. Opt. **34**, 4755–4767 (1995).

24. Mainz, R. E. et al. Phase Stabilization in a Sub-Cycle Parametric Waveform Synthesizer. Ultrafast Optics XII Conference, Talk P1.19 (2019).
